# Characterization of Hydrocarbon Molecular Signatures across Sedimentary Subbasins and Stratigraphic Units: Insights into Source Rock Attributes in Kansas

**DOI:** 10.1021/acsomega.5c11791

**Published:** 2026-07-29

**Authors:** Oluwaseun V. Omoyemi, Henry M. D. Agbogun, James T. Titah, Mohammad W. Rahman, Samuel S. Hanson

**Affiliations:** † Department of Geosciences, Fort Hays State University, Hays, Kansas 67601, United States; ‡ Department of Chemistry, 3901 Tabor College, Hillsboro, Kansas 67063, United States; § Geoscience and Petroleum Research, Inc., Houston, Texas 77084, United States; ∥ School of Arts, Science and Education, Medicine Hat College, Medicine Hat, Alberta T1A 3Y6, Canada

## Abstract

Hydrocarbons constitute an important component of Kansas’s energy sector, but the geochemical characteristics and source rocks of these hydrocarbons are not well understood. This study analyzes 18 hydrocarbon samples from five subbasins and six stratigraphic intervals within the state of Kansas using gas chromatography (GC) and gas chromatography–mass spectrometry (GC-MS) to determine the lithology, sources of organic matter, paleodepositional environment, and thermal maturity of their source rock(s). Analytical results and figures, such as Pr/Ph ratios (≥1), CPI (≥1), low naphthenes/alkanes, low C_29_/C_30_ hopane ratios (<1), and DBT/PHEN vs Pr/Ph plots, suggest a shale lithology for the source rocks. C_19_/C_23_ tricyclic terpane ratios of 0.02–0.32 and sterane/hopane ratios of 0.70–1.08 are observed in hydrocarbons from the Central Kansas Uplift, Hugoton Embayment, Salina Basin, and Sedgwick Basin. In contrast, hydrocarbons from the Forest City Basin display higher C_19_/C_23_ ratios (1.12–2.08) and lower sterane/hopane ratios (0.11–0.13). These geochemical patterns confirm that the hydrocarbons from the former subbasins are derived primarily from marine organic matter, whereas those from the Forest City Basin reflect a dominantly terrigenous source. Paleoenvironmental proxies, including Pr/Ph values (0.98–2.25), C_35_ homohopane index, C_32_/C_31_ hopane ratios (0.47–0.68), and crossplots of Pr/*n*-C_17_ vs Ph/*n*-C_18_, and Gam/C_30_ hopane vs Pr/Ph align with deposition under marginal marine, suboxic conditions with little evidence for water stratification. Thermal maturity proxies (e.g., CPI, OEP, moretane/hopane ratios, C_29_ sterane and C_32_ homohopane isomerization, and Ts/Tm) indicate that the organic matter responsible for these hydrocarbons is within the oil generation window. Overall, the results demonstrate that hydrocarbons from the Forest City Basin were derived from a distinct, terrigenous-rich source rock, whereas the hydrocarbons from the other four subbasins share a genetically related, marine-influenced source. This highlights multiple source rocks for the hydrocarbons produced in Kansas. These findings provide a framework for future studies to consider both local and external sourcing as part of an integrated petroleum system model for Kansas.

## Graphical abstract

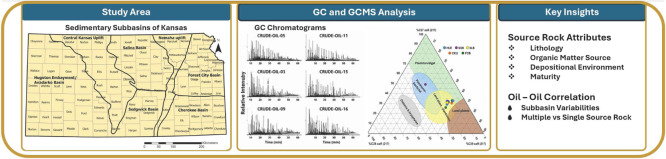

## 1 Introduction

The State of Kansas contributes approximately 1% of the total petroleum production and reserves in the United States, ranking as the 11th largest oil-producing state.^1^ Hydrocarbon production occurs in 87 of the state’s 105 counties and across the different sedimentary basins, which are separated from each other by structural highs.^2,3^ The Central Kansas Uplift (CKU), a prominent structural feature, accounts for the highest hydrocarbon production in the state.^3–5^ Despite significant petroleum production, the characteristics of the hydrocarbons, as well as the petroleum systems within the region, are still poorly understood. Although the reservoir rocks, seals, and traps are well documented,^6–8^ the identity, maturity, source inputs, redox conditions, and paleodepositional environments of the source rock(s) that generated the hydrocarbons being produced remain controversial.^8–10^

Numerous studies worldwide have confirmed that multiple source rocks can contribute to hydrocarbon generation within a single sedimentary basin and stratigraphic column.^11–16^ Conversely, genetically related hydrocarbons have been identified in different sedimentary basins and/or within distinct stratigraphic successions, reflecting a common source rock.^10,17–19^

The uncertainties surrounding the identity and properties of the source rock that generated the hydrocarbons in Kansas complicate the understanding of whether the produced hydrocarbons from different distinct stratigraphic intervals and subbasins share a single or multiple sources.^20,21^ Moreover, the paucity of detailed geochemical descriptions of the produced hydrocarbons limits insights into the petroleum system and its dynamics within Kansas. This invariably impacts the efficiency and optimization of hydrocarbon exploration and production in the state of Kansas. Some studies have reported that the source rocks within Kansas may be thermally immature and unlikely to have generated the observed volume of hydrocarbons.^8–10^ This has led to ongoing debate as to whether the hydrocarbons originated from local sources or migrated from an external source rock. Some researchers argue that sufficiently thick local sources are absent,^9,22–25^ while others propose the presence of local source rock.^26,27^

To address this gap, this study utilizes organic geochemical techniques, such as biomarker analysis, to evaluate the composition of hydrocarbons produced from different subbasins and distinct stratigraphic intervals. Biomarkers are molecular fossils that preserve information about the source, depositional environment, lithology, thermal maturity, and the extent of biodegradation of hydrocarbons, and they are widely used in petroleum geochemistry for their evaluation.^28–34^ This study aims to determine the geochemical signatures of hydrocarbons from different subbasins (Figure 1) and producing intervals (Figure 2) within Kansas and to infer the lithology, source input, maturity, paleoredox conditions, and paleodepositional environment of the source rock(s) that generated them.

**Figure 1:**
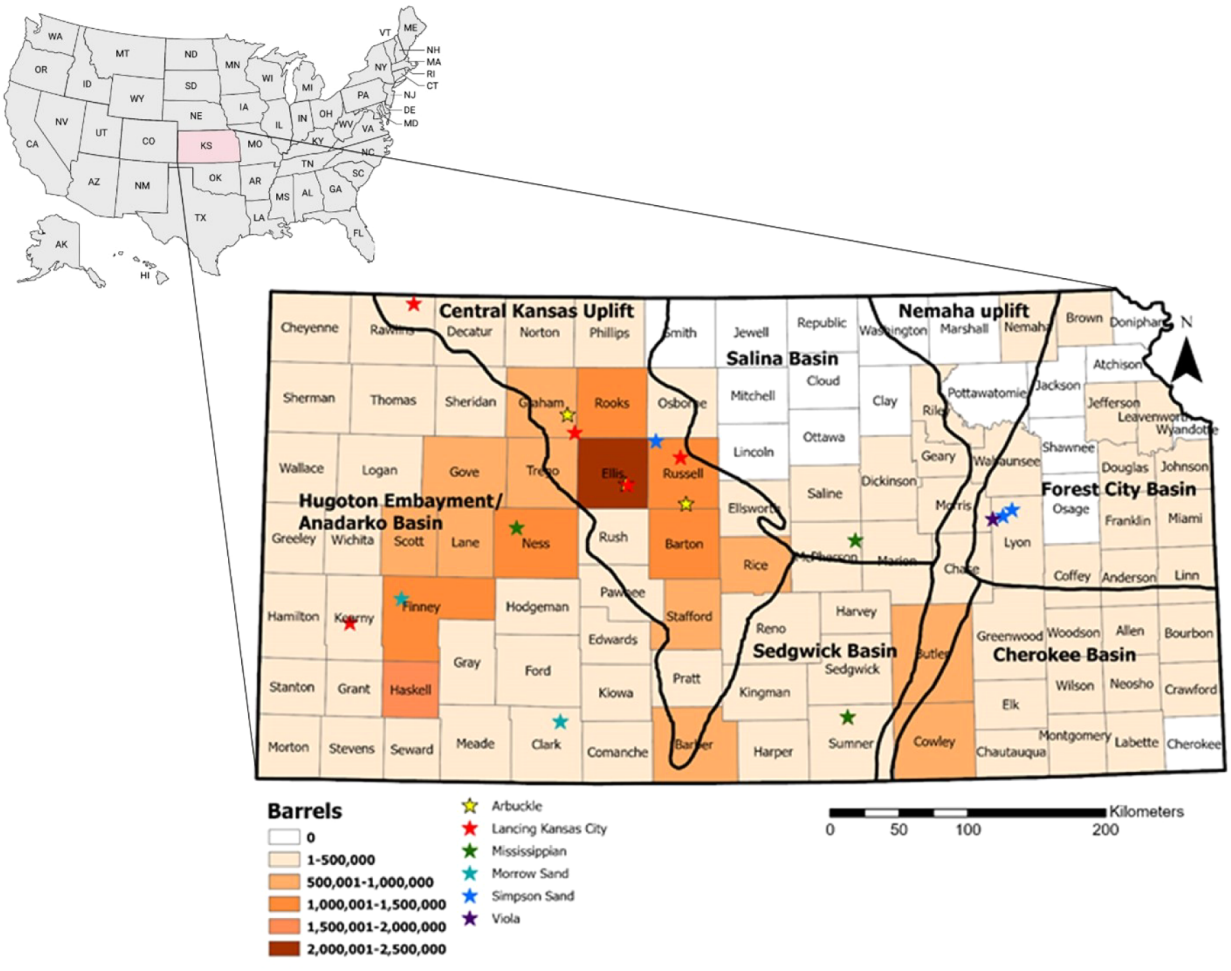
Oil production map of Kansas for 2023, showing the different subbasins and sample locations.

**Figure 2:**
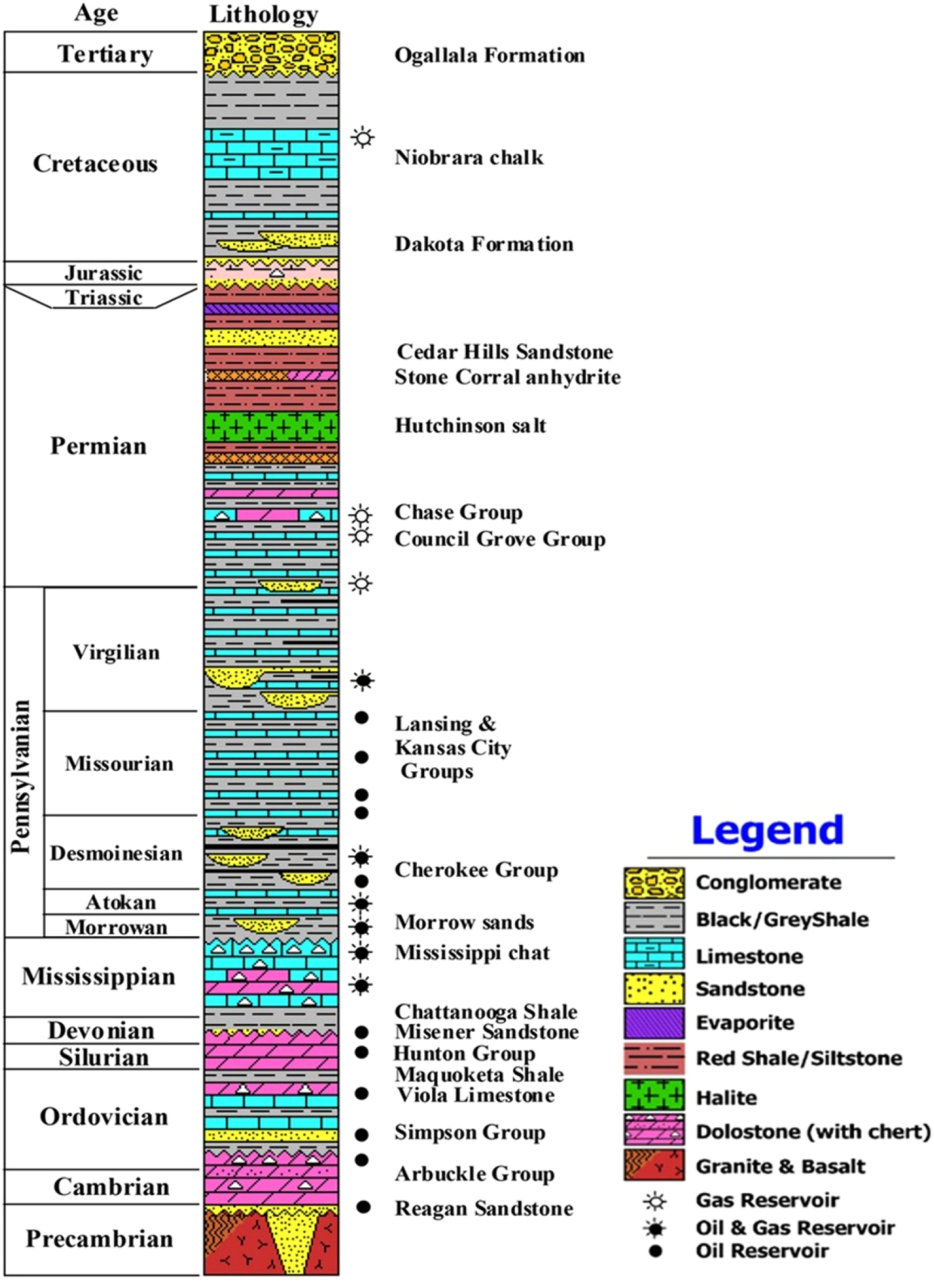
Generalized stratigraphic column of Kansas outlining rock lithology from the Precambrian to the Tertiary geological periods (modified from Doveton, 2017).

These findings will provide a foundation for identifying and defining source rock(s) in Kansas. The results will further clarify whether the produced hydrocarbons are sourced from a single or multiple source rocks, thereby improving our understanding of the petroleum system and its dynamics across the state of Kansas.

## 2 Geological Setting

Kansas consists of Precambrian basement rocks overlain by sedimentary cover.^36,37^ The Precambrian basement represents the continuation of the vast continental core known as the Canadian Shield, visible further north in Canada and in the north-central and northeastern regions of the United States.^4,36,38^ This crystalline basement primarily consists of igneous and metamorphic rocks, such as granite, quartzite, and schist, formed during the Precambrian era.^36,39,40^ Overlying this basement rock is a sedimentary cover, up to approximately 2895 m thick, formed after prolonged nondeposition or erosion of the basement rock at the end of the Precambrian era.^4,5,36^ The generalized stratigraphy is composed mainly of Paleozoic-, Mesozoic-, and Cenozoic-age deposits, including limestone, shale, sandstone, and evaporites^35,36,39^ (Figure 2). These sedimentary layers reflect a variety of ancient marine and terrestrial/terrigenous environments, implying multiple sea-level fluctuations over geological time.^36,39,41^ These sedimentary layers facilitate the exploration of oil and gas, as well as the extraction of valuable underground minerals in Kansas.^36,39,42^

The tectonic framework of Kansas has evolved through multiple phases of deformation, beginning at the Devonian–Mississippian boundary, where major features include the Central Kansas Arch, comprising the Ancestral CKU and Chautauqua Arch, which extends northwest to southeast across the state, along with two subbasins: the Southwest and North Kansas basins.^36,43,44^ A significant structural shift occurred in post-Mississippian to pre-Desmoinesian (Pennsylvanian) time, leading to the full development of the subbasins and present-day features such as the CKU and Nemaha Uplift.^36,44^ Other notable structural features include the Bourbon Arch, Cambridge Arch, Central Kansas Arch, Chautauqua Arch, Las Animas Arch, Ozark Homocline, and Pratt Anticline.^36^

These structural highs shaped regional sedimentation by acting as barriers that influenced differential deposition, thinning, and unconformities.^36,44^

Significant unconformities exist within the Paleozoic, separating the Pennsylvanian from Mississippian rocks, as well as Mississippian-Devonian rocks (Chattanooga Shale) from older Paleozoic formations, reflecting significant periods of erosion and/or nondeposition.^5,45^

Mesozoic rocks in Kansas belong to the Triassic, Jurassic, and Cretaceous geological periods, which unconformably overlie Permian strata. They are predominantly of marine origin, consisting of shale, sandstone, limestone, and chalk, with a few notable occurrences of nonmarine sandstone and clay.^36,40^ While Mesozoic rocks in Kansas do not produce commercial petroleum, other mineral resources, both fuel and nonfuel, have been and continue to be exploited economically.^40,44^

During and after the Cenozoic, an extensive layer of rock debris accumulated over eastern Colorado and western Kansas. The region then experienced an epeirogenic eastward tilt,^5,36,46,47^ forming the Great Plains, essentially representing an eastward-dipping homocline with minor structural irregularities.^36,46,48^

## 3 Samples and Methods

### 3.1 Samples and Sampling

In this study, 18 liquid crude oil samples were collected directly from producing wellheads from five subbasins in Kansas (Hugoton Embayment/Anadarko, Central Kansas Uplift, Salina, Sedgwick, and Forest City basins) and at different producing stratigraphic horizons: Arbuckle, Lansing-Kansas City, Mississippian, Morrow Sand, Simpson Sand, and Viola formations (Figures 1 and 2). These samples were collected from wells with a single producing stratigraphic interval to eliminate the error of commingling from different intervals.

The samples were labeled according to their subbasin, producing interval, and county. The first set of three letters in the sample names represented their subbasin (CKU—Central Kansas Uplift; HUE—Hugoton Embayment; SLB—Salina; SGB—Sedgwick; FCB—Forest City Basin). The second set of three letters in the sample names represented the producing interval from which they were obtained (ARB—Arbuckle; LKC—Lansing Kansas City; MSP—Mississippian; MWS—Morrow Sand; SMS—Simpson Sand; VLA—Viola). The third set of two letters in the sample names represented the counties from which the samples were obtained (EL—Ellis; RS—Russell; RA—Rawlins; GH—Graham; SU—Sumner; MP—McPherson; LY—Lyon; NS—Ness; CA—Clark; FI—Finney; KE—Kearny). All 18 samples were analyzed using gas chromatography (GC), while 14 samples were further examined using gas chromatography–mass spectrometry (GC-MS).

Two CKU samples (CKU-LKC-RS and CKU-LKC-GH), displaying chromatogram patterns very similar to other CKU samples or biodegradation, were excluded from GC–MS analysis to avoid duplication and ensure a balanced sample distribution. Similarly, the FCB-SMS-LY2 sample was excluded based on its GC chromatogram similarity and close spatial proximity to the FCB-SMS-LY4 sample. The CKU-ARB-GH sample was also excluded from GC–MS analysis due to pronounced *n*-alkane stripping in its GC chromatogram, which limits its reliability for biomarker analysis. These screening steps ensured that the final GC-MS data set retained spatial and stratigraphic representativeness while excluding redundant or analytically compromised samples. The analyses conducted in this study were based solely on wellhead oil samples; no source-rock extracts were utilized.

### 3.2 Gas Chromatography

Gas Chromatography (GC) technique was used to analyze and detect the distribution of normal alkanes, as well as biomarkers such as pristane and phytane, present in the hydrocarbon samples. A sample volume between 0.1 and 1.0 μL was injected via a syringe into the split/splitless injector of an Agilent 6890N gas chromatograph, held at 275 °C. The sample was passed through a 60 m × 0.25 mm inner diameter (I.D.) Agilent DB-1 column with helium as the carrier gas under constant pressure. The GC temperature was programmed from 30 °C for 5 min, then increased by 3 °C/min to a final temperature of 320 °C, where it was held for 20 min. Hydrocarbon components eluting from the column were detected by a flame ionization detector (FID) set at 350 °C, allowing quantification of *n*-alkanes and related hydrocarbons in the approximate carbon number range C_4–40_. Compound identification was achieved by comparison of retention times with those of a certified reference material, the Norwegian Oil Standard NGR NSO-1.

### 3.3 Medium-Pressure Liquid Chromatography

Fourteen hydrocarbon samples were fractionated using medium-pressure liquid chromatography (MPLC) to isolate saturate, aromatic, and NSOs (nitrogen, oxygen, and sulfur compounds; resins) compound class fractions for further analysis. An aliquot of each sample was passed through a Sep-Pak Plus CN cartridge before being introduced into the sample injection loops. Once injected, the sample passed through a deactivated silica precolumn, which retains the resin fraction. Prior to chromatographic separation, oils were deasphaltened by the addition of *n*-pentane, which selectively dissolves the maltene fraction (saturates, aromatics, and NSO compounds). The mixtures were thoroughly agitated and subsequently centrifuged to ensure the complete dissolution of maltenes and the quantitative precipitation of asphaltenes, which formed a distinct dark phase at the bottom of the vial.

Samples were eluted sequentially at a constant flow using *n*-hexane. The saturates were collected by an automated fraction collector, the flow of hexane was reversed to the main column, and the aromatics were back-flushed into separation fraction collector tubes. Both refractive index and UV absorption detectors were used to measure the elution of the fractions. The resin fraction was eluted from the precolumn with a 50:50 mix of dichloromethane/methanol using a separate pump.

### 3.4 Gas Chromatography–Mass Spectrometry

Gas chromatography–mass spectrometry (GC-MS) was carried out to analyze the saturated and aromatic biomarkers. Prior to GC-MS analysis, Medium Performance Liquid Chromatography (MPLC) was employed to separate the saturated and aromatic components, allowing for individual analysis of each fraction in biomarker studies. Both the saturated and aromatic fractions were examined using a Hewlett-Packard (HP) 6890 gas chromatograph with an HP 7683 autosampler, on-column injector, and the HP 5973 mass selective detector (MSD). The saturated samples were separated on a 60 m × 0.25 mm inner diameter (I.D.) DB-5 column with a column oven program starting from 100 °C and ending at 320 °C, with a total run time of 118.5 min. Aromatic components were separated using a 60 m DB-1 column, with the column oven program beginning from 85 °C and ending at 325 °C, with a run time of 82.0 min. In both analyses, helium was used as the carrier gas.

The on-column injection technique was used for both the saturated and aromatic GC-MS sample analyses, with the injector temperature programmed at 3 °C above that of the column oven. The MSD was operated in the selected ion monitoring (SIM) mode. For the saturated sample biomarker analysis, the ions were collected twice, from 5 to 30 min and from 30 to 118.5 min, with a dwell time of 20 ms for each ion. Similarly, for the aromatic sample biomarker analysis, the ions were also collected twice, from 5 to 36 min and from 36 to 82 min, with the same dwell time of 20 ms for each ion.

Data acquisition and processing were performed using Agilent ChemStation software. Compound identification was based on retention times in total ion current (TIC) chromatograms and comparison of mass spectra with those of a certified reference material, Norwegian Oil Standard NGR NSO-1. Relative abundances of compound groups within the saturated and aromatic fractions were calculated from the peak areas normalized to the respective internal standards. Results are reported as relative percentages with respect to the total masses of each hydrocarbon fraction.

## 4 Results

### 4.1 Molecular Composition

The gross compositions (Table 1) of the analyzed oils reveal a predominance of saturated hydrocarbons (25.69–68.31%), followed by aromatics (11.93–23.66%), nitrogen, sulfur, and oxygen (NSO) compounds (11.07–28.44%), and asphaltenes (0.76–33.94%). Figure 3 depicts the relative abundance of saturates, aromatics, and NSO compounds with asphaltenes in the samples in a modified ternary plot.^49,50^

**Table 1:** Selected Geochemical Results Derived from Gas Chromatography (GC) and Medium-Performance Liquid Chromatography (MPLC) Analyses of Hydrocarbon Samples^a^

Sample ID	CPI	OEP	NP/AK	Pr/Ph	Pr/*n*-C_17_	Ph/*n*-C_18_	SAT [%]	ARO [%]	NSO [%]	ASPH [%]
CKU-LKC-RS	1.00	1.01	0.17	1.48	0.43	0.41	nd	nd	nd	nd
CKU-LKC-GH	0.98	1.00	0.21	1.41	0.51	0.46	nd	nd	nd	nd
CKU-ARB-GH	0.92	0.83	0.50	0.98	1.86	2.32	nd	nd	nd	nd
CKU-ARB-EL1	0.99	1.00	0.10	1.44	0.49	0.41	55.17	18.33	18.00	8.50
CKU-ARB-RS	1.01	1.00	0.21	1.57	0.36	0.37	60.19	20.19	16.35	3.27
CKU-LKC-EL1	1.01	1.00	0.23	1.58	0.44	0.39	61.30	20.16	16.09	2.44
CKU-LKC-RA	1.00	1.00	0.12	1.52	0.46	0.41	55.56	17.19	18.40	8.85
CKU-SMS-RS	0.99	1.00	0.10	1.48	0.73	0.60	53.51	18.37	20.45	7.67
HUE-LKC-KE	1.03	1.02	0.16	1.80	0.58	0.38	57.33	18.57	18.24	5.86
HUE-MSP-NS1	1.01	1.01	0.09	1.44	0.48	0.39	68.06	16.05	11.56	4.33
HUE-MWS-CA	1.04	1.02	0.18	1.93	0.23	0.21	68.31	17.91	11.22	2.56
HUE-MWS-FI1	1.04	1.01	0.21	1.34	0.68	0.62	51.37	20.89	20.03	7.71
FCB-SMS-LY4	1.09	1.05	0.00	2.25	0.01	0.03	35.76	16.47	23.00	24.78
FCB-VLA-LY	1.08	1.02	0.01	2.06	0.03	0.06	32.65	16.62	25.15	25.59
FCB-SMS-LY2	1.02	1.04	0.01	1.16	0.01	0.04	nd	nd	nd	nd
SLB-MSP-MP	1.01	1.01	0.07	1.40	0.57	0.52	55.11	18.61	20.80	5.47
SGB-LKC-SU	1.06	1.00	0.03	1.12	2.58	2.41	25.69	11.93	28.44	33.94
SGB-MSP-SU	1.00	1.00	0.15	1.53	0.44	0.38	64.50	23.66	11.07	0.76

a **Note:** CPI: carbon preference index, OEP: odd-to-even predominance for *n*-alkanes, NP/AK: naphthenes/alkanes, Pr/Ph: pristane/phytane, SAT: saturated hydrocarbons; ARO: aromatic hydrocarbons, NSO: nitrogen, sulfur, and oxygen, ASPH: asphaltenes, nd: no data.

**Figure 3:**
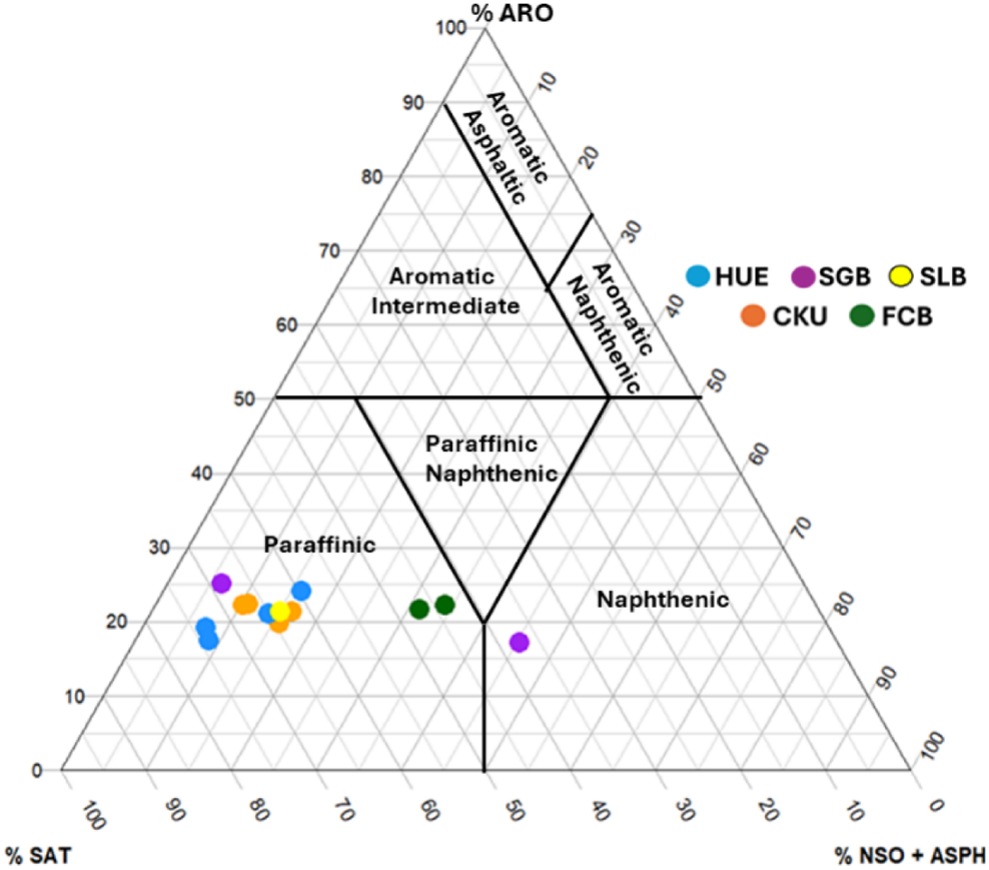
Ternary diagram showing the distribution of SARA fractions (saturates, aromatics, NSO + asphaltenes) of the hydrocarbon samples from the different subbasins, illustrating the relative compositional differences and grouping of the hydrocarbon samples.

#### 4.1.1 *n*-Alkanes Distribution

The distribution of *n*-alkanes in the samples analyzed reveals carbon atom series ranging from C_4–40_. The C_5–12_ hydrocarbon fractions are classified as light hydrocarbons, and the C_13–40_ fractions are classified as heavy hydrocarbons.^50–52^ The relative distributions of light and heavy hydrocarbons in the samples analyzed are presented in Figure 4. The heavy hydrocarbon fractions dominate (more than 50%) in all samples from the HUE, FCB, SGB, and SLB, while light hydrocarbon fractions dominate in four of the eight samples from the CKU. The whole gas chromatograms of the analyzed hydrocarbon samples, highlighting the *n*-alkane and isoprenoid distribution patterns, are presented in Figure 5. The shape and distribution of compounds in the chromatograms vary among the different samples. Some of the samples analyzed show unimodal normal alkane distribution patterns, while others display bimodal patterns (Figure 5). However, low-molecular-weight *n*-alkanes are depleted in samples CKU-ARB-GH and SGB-LKC-SU, with a preferential removal of *n*-alkanes relative to isoprenoids. The FCB samples are dominated by odd-to-even *n*-alkanes (C_11–19_ range), minimal C_20+_ components, and a near absence of isoprenoids.

**Figure 4:**
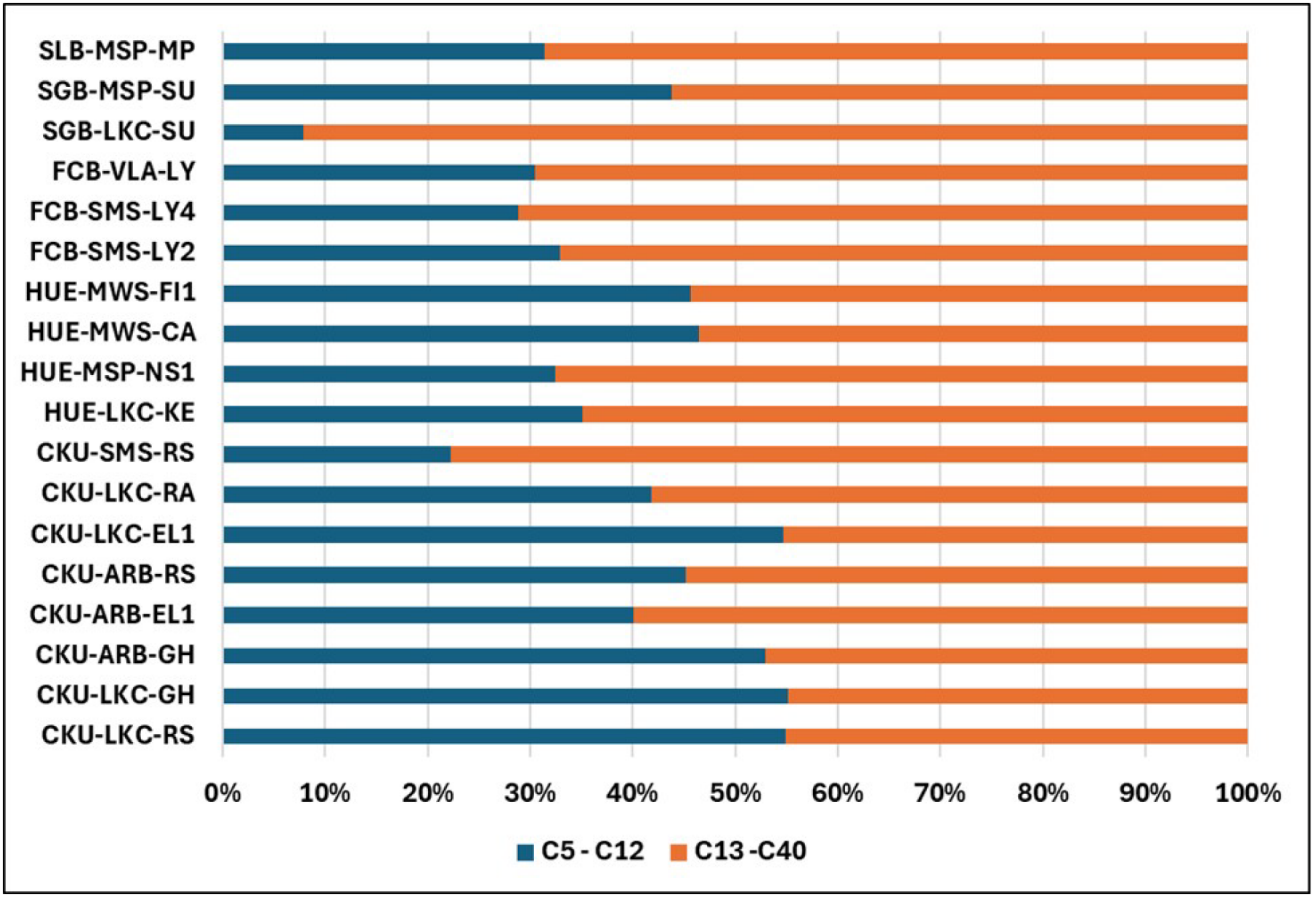
Distribution of light (C_5–12_) and heavy (C_13–40_) hydrocarbon fractions in the analyzed samples, showing the dominance of heavy fractions in SLB, SGB, FCB, and HUE subbasins.

**Figure 5:**
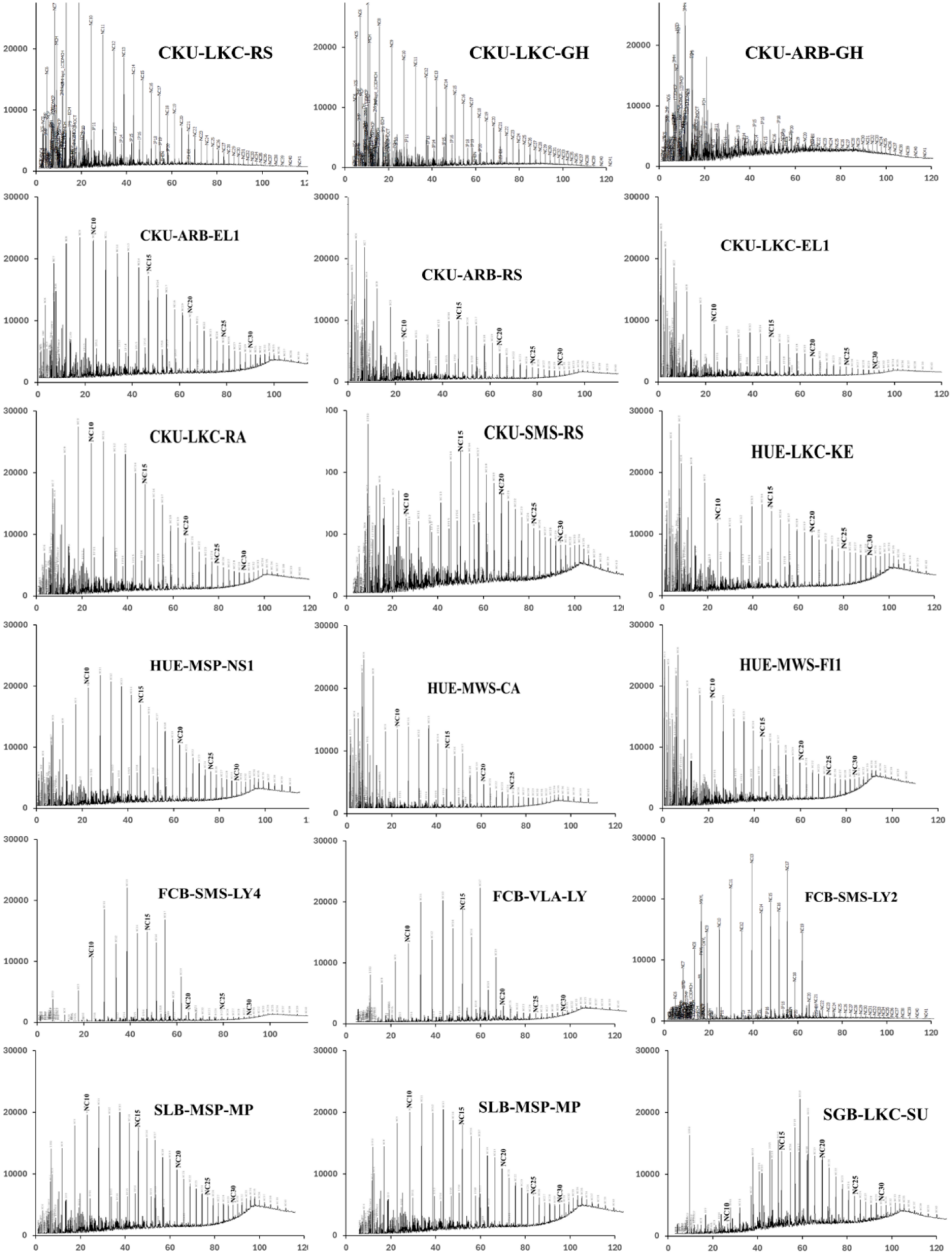
Gas chromatograms of oil samples analyzed, highlighting variations in the distribution of *n*-alkanes (the *x*-axis represents retention time, and the *y*-axis represents relative abundance; NC represents the number of carbon atoms in the *n-*alkanes).

The carbon preference index (CPI), which is commonly used to evaluate both source lithology and thermal maturity,^32,53–55^ ranged from 0.92 to 1.09 (Table 1). The naphthenes-to-alkanes (NP/AK) ratio, which describes the relative abundance of cyclic to linear saturated hydrocarbons, was also evaluated. Calculated NP/AK ratios ranged from 0.00 to 0.50 (Table 1), implying low proportions of cyclic hydrocarbons across most samples.

#### 4.1.2 Biomarkers

The ratio of pristane/phytane (Pr/Ph) is widely used for evaluating depositional environments and organic matter sources.^50,52,56–58^ An inspection of the chromatograms reveals substantially lower concentrations of both pristane and phytane in the FCB samples compared to the other samples (Figure 5). Across the data set, pristane is consistently more abundant than phytane, with Pr/Ph ratios ranging from 0.98 to 2.25 (Table 1). The Pr/*n-*C_17_ and Ph/*n-*C_18_ ratios are similar in most oils (Pr/*n*-C_17_ avg. 0.49 ± 0.13 and Ph/*n-*C_18_ avg. 0.43 ± 0.11); however, the FCB samples exhibit distinctly lower values (Pr/*n*-C_17_ avg. 0.02 ± 0.01 and Ph/*n-*C_18_ avg. 0.04 ± 0.02), while the CKU-ARB-GH and SGB-LKC-SU samples display elevated ratios (Pr/*n*-C_17_ avg. 2.22 ± 0.51 and Ph/*n-*C_18_ avg. 2.36 ± 0.06).

Gammacerane is a biomarker commonly linked to stratified water columns in both marine and nonmarine source-rock depositional environments, often linked with hypersaline conditions.^33,59^ Gammacerane is detected in very low amounts in all samples analyzed from the different subbasins and producing intervals, with gammacerane/C30 hopane ratios ranging from 0.07 to 0.28 (Table 2). The dibenzothiophene/phenanthrene (DBT/PHEN) ratio, used to infer the source rock lithology and depositional environment,^60^ ranges from 0.10 to 0.65 (Table 2).

**Table 2:** Geochemical Results Derived from GC-MS Analysis of Hydrocarbon Samples^a^

Sample ID	a [%]	b [%]	c [%]	d	e	f	g	h	I	j	k	l	m	n	o
CKU-ARB-EL1	30.96	15.17	53.87	0.47	0.57	0.60	0.37	0.10	0.78	0.06	0.65	0.09	0.61	0.86	0.63
CKU-ARB-RS	30.10	12.70	57.20	0.48	0.57	0.59	0.39	0.10	0.75	0.08	0.32	0.08	0.61	0.69	0.59
CKU-LKC-EL1	31.12	16.06	52.82	0.50	0.56	0.61	0.41	0.10	0.94	0.07	0.45	0.07	0.59	0.75	0.59
CKU-LKC-RA	30.06	16.58	53.35	0.47	0.56	0.62	0.40	0.10	0.88	0.06	0.49	0.07	0.62	0.65	0.58
CKU-SMS-RS	29.11	17.53	53.36	0.44	0.58	0.59	0.39	0.09	0.85	0.07	0.39	0.08	0.58	0.78	0.61
FCB-SMS-LY4	25.95	20.13	53.92	0.61	0.52	0.61	0.29	0.09	0.13	2.08	0.10	0.07	0.54	0.67	0.60
FCB-VLA-LY	32.83	11.09	56.08	0.47	0.50	0.59	0.23	0.12	0.11	1.12	0.11	0.08	0.57	0.57	0.65
HUE-LKC-KE	28.89	17.63	53.49	0.46	0.57	0.59	0.36	0.09	0.75	0.12	0.25	0.08	0.56	0.71	0.68
HUE-MSP-NS1	31.98	14.82	53.20	0.51	0.60	0.62	0.60	0.10	1.08	0.05	0.29	0.08	0.63	0.70	0.51
HUE-MWS-CA	32.60	10.50	56.90	0.47	0.59	0.60	0.47	0.10	0.70	0.32	0.16	0.10	0.57	1.12	0.68
HUE-MWS-FI1	32.44	15.83	51.73	0.53	0.56	0.60	0.35	0.11	0.82	0.08	0.50	0.09	0.60	0.82	0.62
SGB-LKC-SU	49.09	26.14	24.77	0.53	0.41	0.64	0.42	0.16	0.86	0.02	0.16	0.28	0.64	0.71	0.47
SGB-MSP-SU	31.81	15.57	52.62	0.49	0.64	0.61	0.50	0.09	1.01	0.09	0.10	0.14	0.59	0.85	0.59
SLB-MSP-MP	30.72	16.47	52.81	0.51	0.57	0.62	0.31	0.09	0.81	0.05	0.41	0.10	0.62	0.82	0.58

a **Note:** a: % C_27_ regular steranes, b: % C_28_ regular steranes, c: % C_29_ regular steranes, d: C_29_ 20S/(20S + 20R) sterane, e: C_29_ ββ/(ββ + αα) sterane, f: C_32_ homohopane 22S/(22S + 22R), g: Ts/(Ts + Tm) Ts C_27_ 18α­(H)-22,29,30-trisnorneohopane and Tm (C_27_ 17α­(H)-22,29,30-trisnorhopane, h: Moretane/hopane, i: Steranes/hopanes, j: C_19_/C_23_ tricyclic terpanes, k: Dibenzothiophene/phenanthrene (DBT/PHEN), l: Gammacerane/C30 hopane, m: Norhopane/hopane (C_29_/C_30_), n: C_35_ Homohopane index (C_35_/C_34_), o: C_32_ homohopane/C_31_ homohopane (C_32_H/C_31_H).

The C_27–29_ ααα R isomers, regular steranes, are present in all samples. These steranes are typically associated with different sources of organic matter.^34,61^ The relative abundance of C_27_, C_28_, and C_29_ regular steranes in all samples analyzed from the different subbasins and producing intervals is shown in Table 2, with C_27_ sterane ranging from 25.95% to 49.09%, C_28_ from 10.50% to 26.14%, and C_29_ from 24.77% to 57.20%.

Sterane and terpane isomerization indices, such as C_29_ sterane 20S/(20S + 20R), C_29_ sterane ββ/(ββ + αα), C_31_ or C_32_ homohopane 22S/(22S + 22R), and Ts/(Ts + Tm), serve as strong maturity indicators.^34,62–65^ The C_29_ sterane 20S/(20S + 20R) ratio ranged from 0.44 to 0.61, while the C_29_ sterane ββ/(ββ + αα) ratio ranged from 0.41 to 0.64 (Table 2). The C_32_ homohopane 22S/(22S + 22R) ratio varied from 0.59 to 0.64, and the Ts/(Ts + Tm) ratios ranged from 0.23 to 0.60 (Table 2). The moretanes/hopanes ratio, which decreases with thermal maturity,^63,66^ ranged from 0.09 to 0.16 across the samples (Table 2).

## 5 Discussion

### 5.1 Lithology

The combination of biomarker and nonbiomarker data can effectively distinguish carbonates and shale source rocks.^32,34,50,53,54^ Hydrocarbons generated by nonmarine source rocks can be differentiated from those derived from marine shale or marine carbonate sequences using various geochemical signatures.^32,34,53^ Steranes, *n*-alkanes, isoprenoids, and terpanes are reliable biomarkers for determining source rock lithology.^32,34^ As such, the lithology of the source rock(s) that generated the hydrocarbons analyzed in this study can be inferred from GC and GC-MS results.

Shale source rocks are typically known to have high (≥1 to <3) pristane/phytane (Pr/Ph) ratios.^32,53,54,67,68^ The analyzed samples exhibit high Pr/Ph ratios ranging from 0.98 to 2.25 (Table 1), supporting a shale lithology for their source rock. Naphthenes-to-alkanes (NP/AK) ratios have been determined to range from low to medium for shale source rocks.^50,53^ The NP/AK ratios for the analyzed samples are low, ranging from 0.00 to 0.50 (Table 1), thereby reinforcing an interpretation of shale lithology for their source rock. The carbon preference index (CPI), with values greater than or equal to one (≥1), is generally interpreted to be from source rocks with shale lithology.^32,53,54,67^ The CPI values of 0.92 to 1.09 (Table 1) for the analyzed samples therefore signify a shale lithology for the source rock of the hydrocarbon samples.

The dibenzothiophene/phenanthrene (DBT/PHEN) ratio is another key approach used for evaluating lithology.^53,60^ When the DBT/PHEN ratio is plotted against Pr/Ph on the Hughes diagram,^60^ the analyzed oil samples fall within Zone 3, validating the inference of shale lithology as the source rock that generated the hydrocarbons (Figure 6).

**Figure 6:**
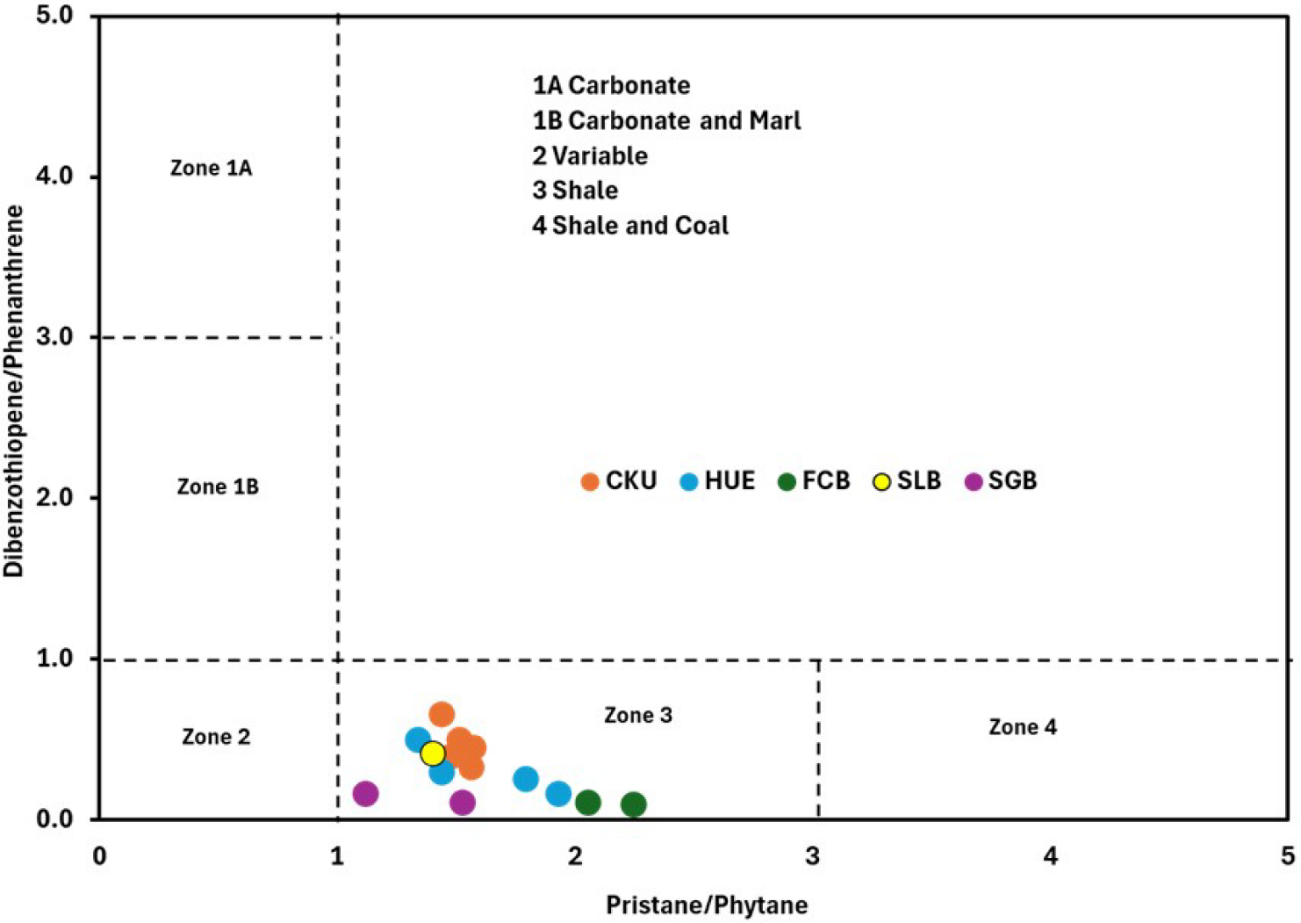
DBT/PHEN vs Pr/Ph ratio signifying shale lithology as the source rock that generated the hydrocarbon samples.

Furthermore, C_22_/C_21_ and C_24_/C_23_ tricyclic terpane ratios help distinguish extracts and crude oils derived from clay-rich (shale) vs carbonate source rocks.^34,69^ High C_22_/C_21_ and low C_24_/C_23_ tricyclic terpane values are widely recognized in carbonate source rocks, whereas low C_22_/C_21_ and high C_24_/C_23_ values are indicative of shale-derived source rocks. The samples analyzed display C_22_/C_21_ and C_24_/C_23_ ratios ranging from 0.25 to 0.40 and 0.41–0.81, respectively (Table 2). The C_22_/C_21_ vs C_24_/C_23_ plot shows that the samples fall within the shale source-rock field, except for sample SGB-LKC-SU, which plots outside the main cluster (Figure 7). Although the SGB-LKC-SU hydrocarbon plots outside the main cluster, its elevated C_24_/C_23_ relative to C_22_/C_21_ tricyclic terpane ratios support a shale rock derivation.

**Figure 7:**
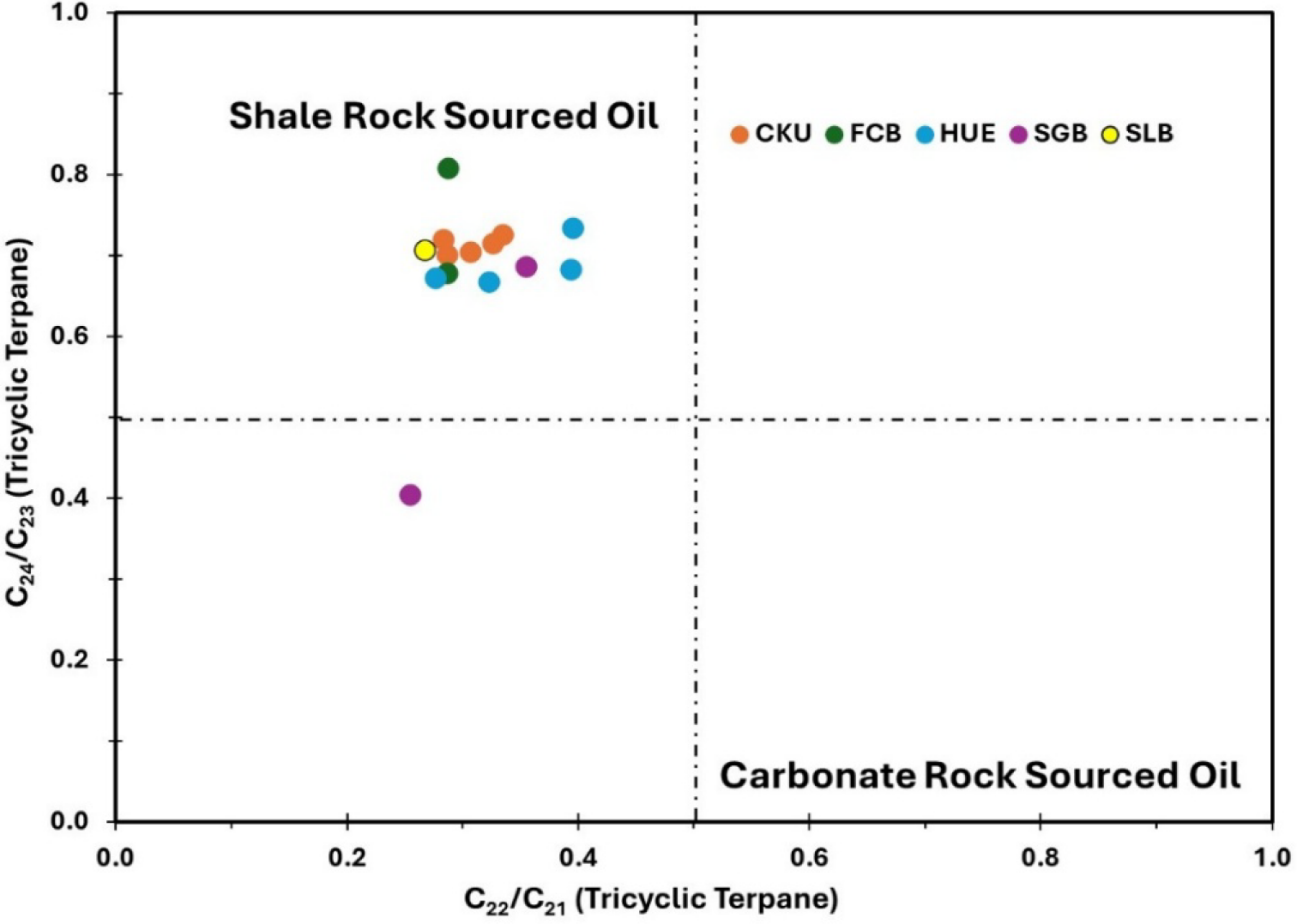
Crossplot of C_22_/C_21_ vs C_24_/C_23_ tricyclic terpanes, illustrating a shale source rock for the hydrocarbon samples from the different subbasins and producing intervals.

Additionally, a low norhopane/C_30_ hopane (C_29_/C_30_) ratio, typically <1, has been reported to be diagnostic of shale source rocks.^68,70^ The samples analyzed in this study have low C_29_/C_30_ hopane ratios ranging from 0.54 to 0.64 (Table 2), which correlates well with a shale lithology source rock. The multiple parameters used to interpret the lithology in this study all validated each other. It can therefore be concluded that they are all generated from source rock(s) with a shale lithology, irrespective of their stratigraphic interval or subbasins.

### 5.2 Organic Matter Source

Organic matter in source rocks can be sourced from marine or terrigenous environments and can also be a mixture of both environments.^33,34^ The source of organic matter influences the properties of the hydrocarbons they generate.^33,34^ As such, the geochemical signatures of hydrocarbons can be used to infer the source of organic matter that generated them. However, it is well established that organic matter derived from multiple sources, sediment reworking, and long-distance transport can significantly modify (such as through biodegradation, and water washing) the original geochemical signatures preserved in oils.^33,50,71–73^ Multisource mixing may produce composite biomarker and isotopic signals, while the incorporation of reworked or allochthonous organic matter can introduce maturity and compositional discrepancies that are unrelated to the in situ depositional environment. In addition, long-distance fluvial or marine transport can relocate terrestrial or marine organic matter far from its site of production, further complicating interpretation.^34^ These processes can lead to ambiguous or even erroneous interpretations of organic matter provenance if not carefully evaluated. To minimize such uncertainties, we applied a multiparameter geochemical approach to constrain the source of organic matter in the analyzed samples. The use of multiple independent proxies enhances the robustness of source interpretation and reduces the likelihood of misattribution arising from mixing, reworking, or long-distance migration effects. Regular steranes (C_27–29_ ααα R isomers) are widely employed tools for evaluating organic matter input, despite some inconsistencies in their application.^32,52,61,74^ C_27_ steranes are predominantly sourced from marine planktonic algae, C_28_ steranes from marine diatoms and lacustrine algae, and C_29_ steranes from higher plants.^61,62,75^ To assess the relative quantity of the C_27–29_ ααα R isomers in the samples analyzed, a ternary diagram was plotted and interpreted using the Huang and Meinschein (1979) approach (Figure 8). The analyzed samples all plot in a cluster, denoting a mixture of marine plankton and land plant organic matter, except for one sample from the SGB. The lone sample (SGB-LKC-SU) plotting outside the cluster may be ascribed to sourcing from marine plankton organic matter. However, considering that it contains a low amount of C_29_ fractions, which might be due to biodegradation, its interpretation is not considered reliable. The high asphaltene fraction (33.94%, Table 1) in the sample serves as evidence of the sample being likely biodegraded.^76,77^ An inspection of its chromatogram (Figure 5) records a reduction in its C_8–12_ *n-*alkane which is compatible with biodegradation.^78^ We therefore conclude that biodegradation has influenced its biomarker distribution, making its interpretation less reliable

**Figure 8:**
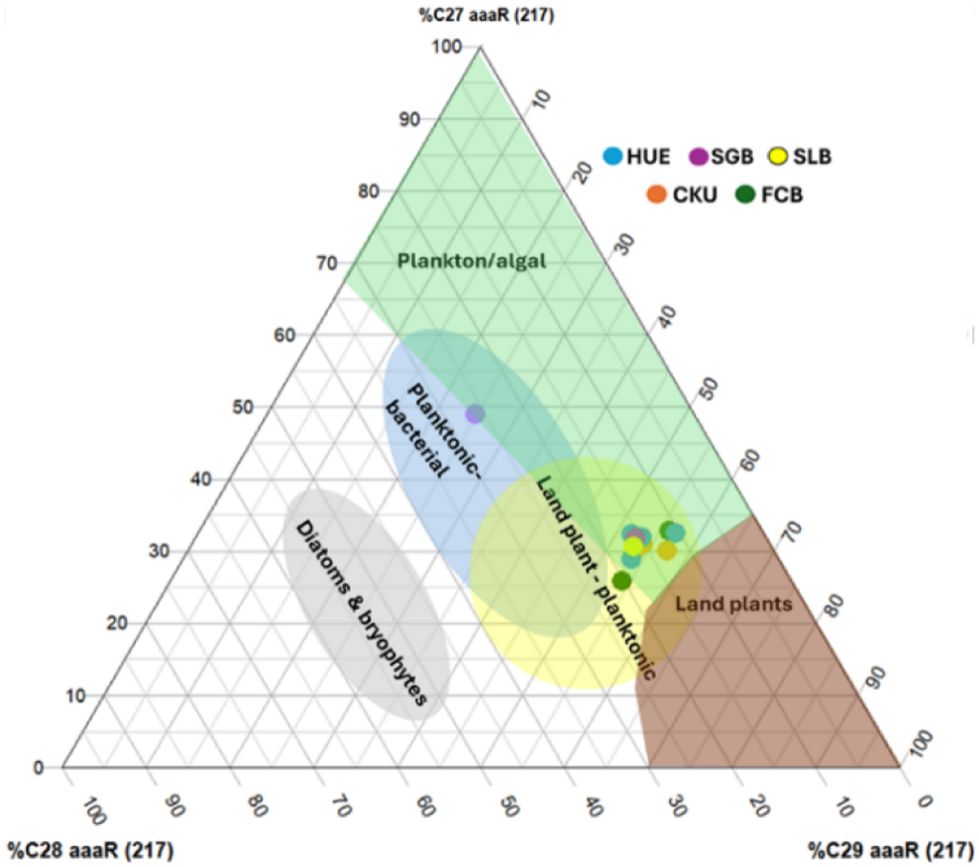
Ternary diagram of the relative abundances of C_27_, C_28_, and C_29_ regular steranes, denoting organic matter sourcing from marine plankton and land plant organic matter.

Sterane-to-hopane ratio also provides insight into the organic matter source.^32,50^ High ratios (≥1) typically denote marine sources with contributions from planktonic and/or benthic algae, whereas low values are correlated with terrigenous input and/or microbial reworking of organic matter.^32,50^ The sterane-to-hopane ratios for the samples from the CKU, HUE, SLB, and SGB subbasins were approximately 1, signifying marine-sourced organic matter (Table 2). However, the samples from the FCB subbasins exhibited ratios of 0.11 and 0.13, compatible with either terrigenous-sourced organic matter or products of organic matter reworking.

Tricyclic terpane distributions have also been used by numerous studies to differentiate organic matter sourcing.^33,79,80^ The C_19_ and C_20_ tricyclic terpanes are reported to primarily originate from higher plants, while C_21_+ tricyclic terpanes are mainly derived from bacteria and algae.^80^ Low C_19_/C_23_ tricyclic terpane ratio commonly occurs in marine organic matter input, whereas higher values (≥1) are observable in terrigenous organic matter input.^33^ Results from samples analyzed in this study have C_19_/C_23_ ratio ranging from 0.02 to 0.32 for samples from the CKU, HUE, SLB, and SGB, while the FCB samples ranged from 1.12 to 2.08. These results correspond to marine-sourced organic matter for all samples, except for the FCB samples, which are interpreted to be terrigenous-sourced. The SMS sample from the CKU differs in organic matter source relative to the FCB samples, thereby emphasizing how geographic variation governs organic matter contribution across stratigraphic intervals.

Analysis of the ternary distribution of C_27_, C_28_, and C_29_ regular steranes initially indicates a mixed input of marine and terrestrial organic matter in the analyzed samples. However, additional biomarker data analysis provides a clearer source discrimination. Specifically, sterane/hopane ratios and C_19_/C_23_ tricyclic terpane ratios distinctly differentiate the samples, with those from CKU, HUE, SLB, and SGB exhibiting properties consistent with predominantly marine-sourced organic matter, whereas the FCB samples display signatures of a terrigenous source. Therefore, on the basis of the various geochemical criteria used in inferring the sources of the organic matter that generated the analyzed samples, it is concluded that the hydrocarbons in Kansas are generated by marine and terrigenous organic matter. The differences in organic matter sourcing are a function of the subbasins and not the producing intervals. Furthermore, the uniquely different organic matter sources interpreted for the FCB may be attributed to its geographic location in the northeastern corner of Kansas. The subbasin may have received sediments and organic matter from the northeast of Kansas that were not readily available to other parts.

### 5.3 Depositional Environment and Redox Conditions

The redox conditions and environment in which organic matter is deposited influence the characteristics of the hydrocarbons they generate.^32,56,68,81^ As such, geochemical assessments of hydrocarbons can be used to provide insights into the depositional environment and redox conditions of their source rocks. The pristane/phytane (Pr/Ph) ratio is a widely applied proxy for assessing depositional redox conditions.^52,56–58^ Pr/Ph values <2 are typically linked to marine organic matter under reducing environments, while values >3 are reported for terrigenous organic matter under oxidizing conditions.^33,34^ The Pr/Ph ratios of the samples analyzed in this study ranged from 0.98 to 2.25 (Table 1), aligning with deposition under reducing conditions within a marine or marginal-marine environment. The Pr/*n*-C_17_ vs Ph/*n*-C_18_ plot has also been used to infer source rock depositional environments and redox conditions,^82,83^ with its reliability grounded in the implications of Pr/Ph ratios for sedimentary facies.^84^ In this study, the crossplot of Pr/*n*-C_17_ vs Ph/*n*-C_18_ places the samples within a depositional setting defined by reducing conditions in a marginal marine environment (Figure 9). However, the FCB samples have very low Pr/*n*-C_17_ (avg. 0.02 ± 0.01) and Ph/*n*-C_18_ (avg. 0.04 ± 0.02) ratios, due to the depletion of their isoprenoids, as observed in Figure 5. As a result, their depositional environment cannot be reliably interpreted.^85,86^ The Pr/*n*-C_17_ vs Ph/*n*-C_18_ crossplot (Figure 9) additionally provides a basis for assessing biodegradation, with ratios exceeding 1 commonly reported for biodegraded hydrocarbons.^51,87^ In this study, both CKU-ARB-GH and SGB-LKC-SU ratios were above 1 (Table 1), likely evidence of biodegradation. The higher Pr/*n*-C_17_ and Ph/*n*-C_18_ ratios observed in samples CKU-ARB-GH and SGB-LKC-SU (Table 1) are ascribed to the preferential removal of *n*-alkanes compared to isoprenoids, as isoprenoids are more resistant to biodegradation than *n*-alkanes.^88,89^ The chromatograms (Figure 5) reveal that the SGB-LKC-SU retains higher *n*-alkane concentrations relative to isoprenoids than the CKU-ARB-GH, representing varying degrees of biodegradation. Accordingly, CKU-ARB-GH is classified as moderately biodegraded, whereas SGB-LKC-SU is classified as slightly biodegraded.^34,88^ Based on the observed biodegradation in the CKU-ARB-GH and SGB-LKC-SU samples, they have been generally excluded from our interpretations.

**Figure 9:**
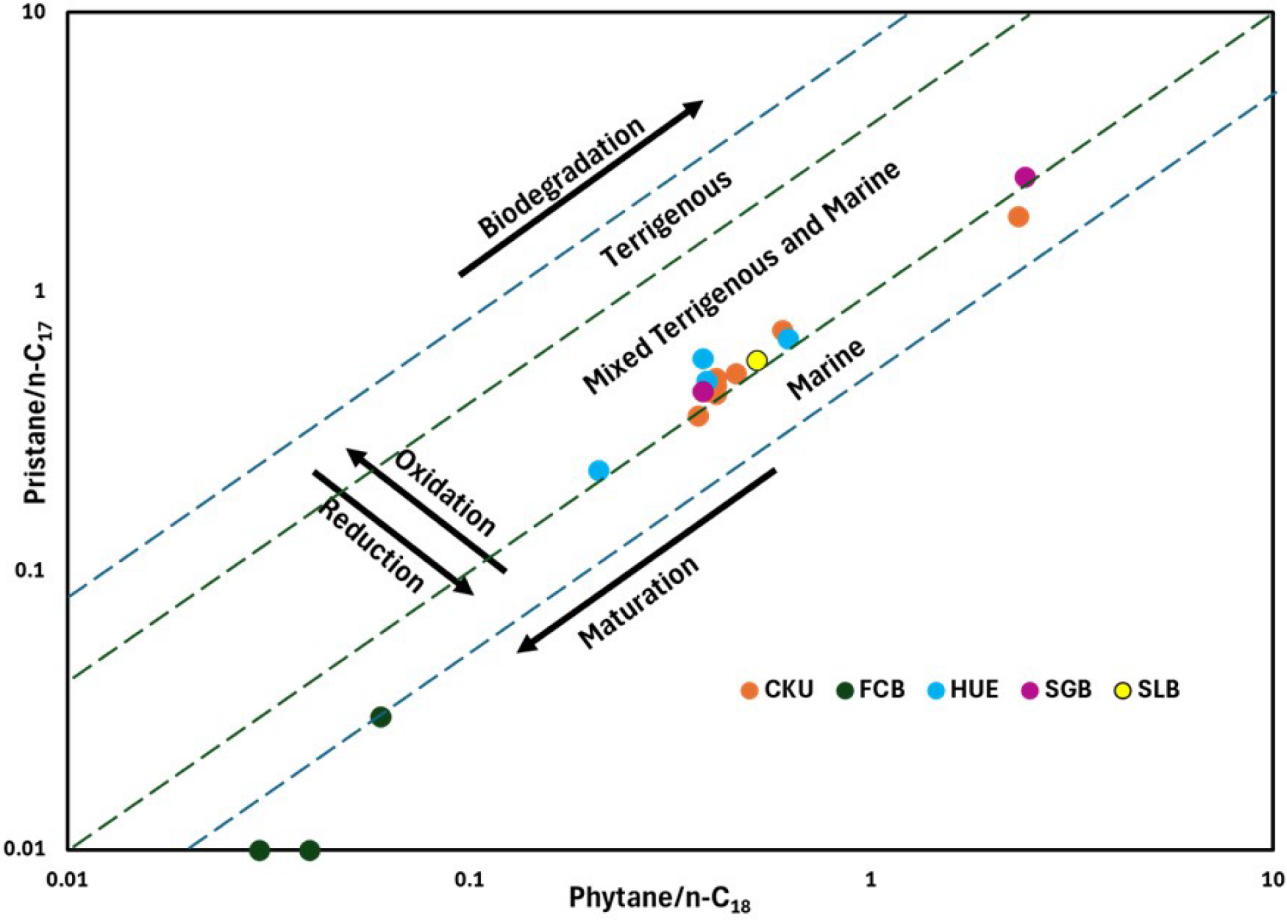
Crossplot of Pr/*n*-C_17_ vs Ph/*n*-C_18_ demonstrating mixed terrigenous and marine sources for the organic matter.

The C_35_ homohopane index, commonly expressed as the C_35_/C_34_ ratio, serves as a proxy for describing redox potential (Eh) in the source rock depositional environment.^34,86,90^ High C_35_/C_34_ values (> 0.8) correspond to highly reducing (low Eh) anoxic conditions, whereas low C_35_/C_34_ (<0.6) occurs under more oxic depositional conditions.^86,90^ The C35/C34 ratios of samples analyzed ranged from 0.57 to 1.12 (Table 2), spanning redox conditions from suboxic to anoxic. However, the C_35_ homohopane index is documented to be greatly influenced by maturity and biodegradation;^90–92^ as such, this study further explored the use of the C_32_H/C_31_H ratio, which is more resistant to maturity and biodegradation.^93^ Anoxic conditions can lead to the reduction of the carboxyl group of C_32_ hopanoic acids to C_32_ homohopanes and oxidation to C_31_ homohopane under oxic conditions.^93^ The C_32_H/C_31_H ratios in the samples analyzed ranged from 0.47 to 0.68, demonstrating deposition under suboxic conditions.

To further elucidate the redox conditions of the environment of deposition of the source rock of the samples, gammacerane, a diagnostic biomarker of a stratified water column commonly occurring under hypersaline conditions,^33,59,94^ was examined. However, water column stratification can also result from temperature gradients.^34^ The primary source of tetrahymanol, a precursor to gammacerane, appears to be bacterivorous ciliates that thrive at the interface between oxic and anoxic zones in stratified systems.^59^ Consequently, a high gammacerane concentration is widely regarded as evidence for the presence of a stratified water column and potentially salinity stratification during sediment deposition.^34,59^ A crossplot of the Gam/(Gam + C_30_ Hopane) ratio vs Pr/Ph places most samples in the dysoxic to suboxic zone (Figure 10), signifying deposition under low-oxygen conditions with normal marine salinity and the absence of water column stratification (based on the very low amount of gammacerane in the samples). However, the SGB samples plotted within the enhanced salinity field may be attributable to temperature effects rather than high water salinity.

**Figure 10:**
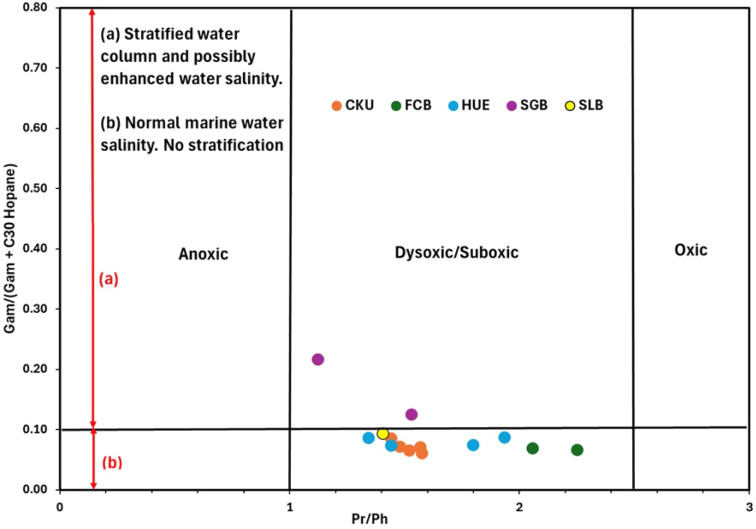
Plot of Gam/(Gam + C_30_ hopane) ratio vs Pr/Ph, illustrating a dysoxic/suboxic condition for the source rock that generated the oils, with normal marine salinity and minimal water column stratification.

### 5.4 Thermal Maturity

Thermal maturity describes the extent of heat-driven reactions that transform sedimentary organic matter into petroleum.^34^ Organic matter is classified as immature, mature, or postmature based on its position relative to the oil-generative window.^50^ Several nonbiomarker parameters are commonly used to assess thermal maturity.^34,55,95^ The ratio of specific *n*-alkanes, such as the carbon preference index (CPI)^55^ and odd-to-even predominance (OEP),^95^ provides information on maturity but may also be influenced by source attributes such as organic matter input and biodegradation.^34^ The CPI and OEP values of 1.0 are reported to denote generation from thermally matured organic matter, while values below 1.0 denote generation from low-thermally matured organic matter.^34,55,95^ The CPI and OEP values for the samples analyzed were approximately 1.0 (excluding the CKU-ARB-GH sample, which was interpreted to be biodegraded), suggesting that they were all generated from thermally matured organic matter.

Biomarker-based thermal maturity metrics, such as the ratios of steranes and terpanes, have been widely used to infer maturity.^34,63,64,66^ The isomerization of steranes (e.g., C_29_ sterane 20S/(20S + 20R); C_29_ sterane ββ/(ββ + αα)) and terpanes (e.g., C_31_ or C_32_ homohopane 22S/(22S + 22R)), as well as Ts/(Ts + Tm) and tricyclic terpane ratios, are well-established tools for assessing thermal maturity.^34,62–65^ The sterane isomerization ratio 20S/(20S + 20R) is particularly diagnostic for distinguishing immature to mature stages of oil, and this is most commonly reported for C_29_ steranes due to the ease of analysis via *m*/*z* 217 mass chromatograms.^34^ Isomerization ratios for C_27_ and C_28_ steranes often suffer from interference due to coeluting peaks.^34^ The ββ/(ββ + αα) isomerization ratio of C_29_ sterane is also highly specific for assessing immature to mature samples.^34^ The 20S/(20S + 20R) and ββ/(ββ + αα) ratios of C_29_ sterane increase with maturity until equilibrium is attained at approximately 0.55 and 0.71, respectively.^34,64^ The thermal maturity of the source rock that generated the analyzed samples is assessed by the crossplot of ββ/(ββ + αα) vs 20S/(20S + 20R) of their C_29_ steranes, as a function of their subbasins (Figure 11). The samples analyzed all plot within the thermally mature zone, irrespective of their intervals or subbasins. This suggests all hydrocarbons were generated from source rock(s) composed of thermally matured organic matter.

**Figure 11:**
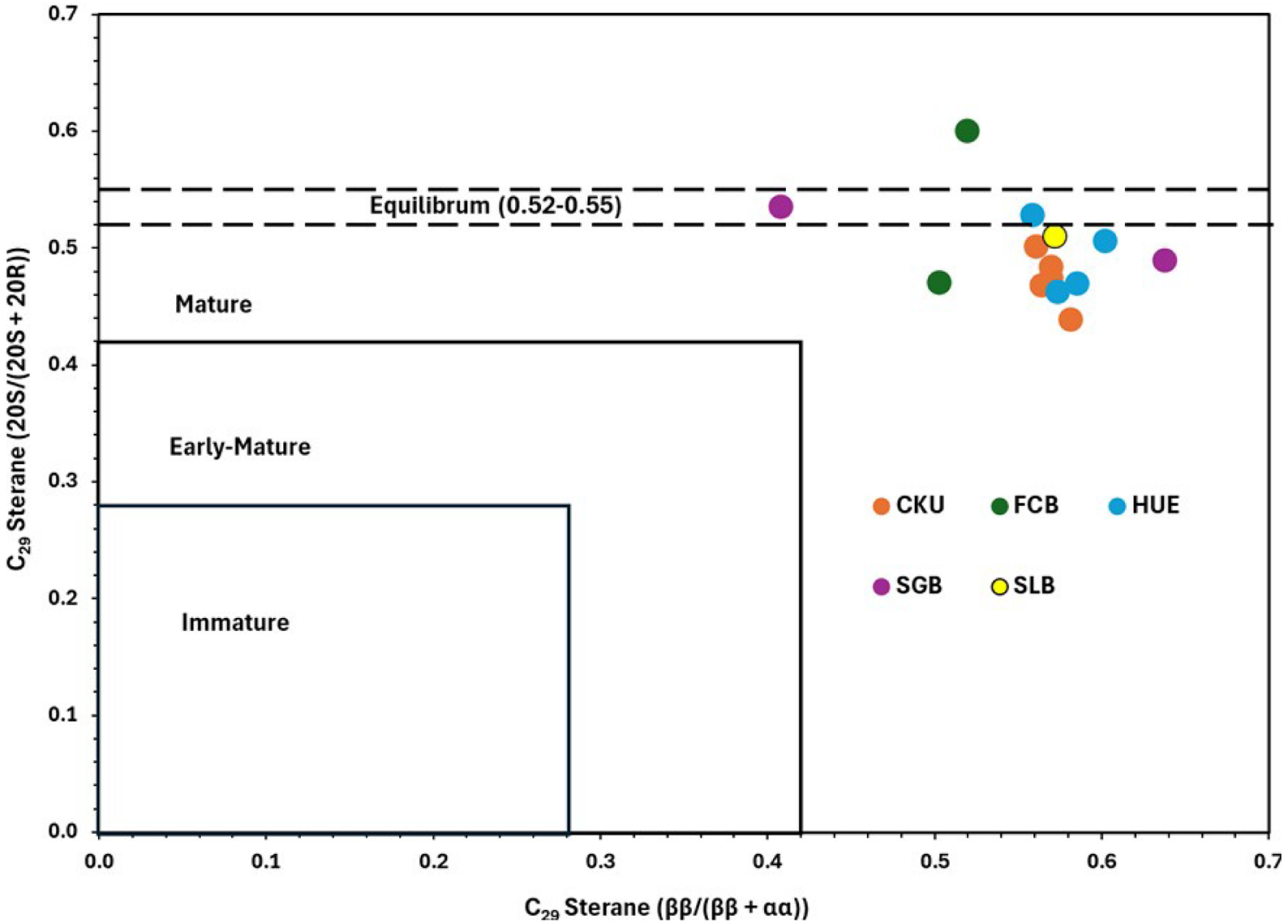
Crossplot of C_29_ sterane 20S/(20S + 20R) ratio and C29 ββ/(ββ + αα) ratio, depicting the thermal maturity of the analyzed hydrocarbons.

The 22S/(22S + 22R) ratio in the C_31–C35_ homohopanes is also commonly used to assess the thermal maturity of source rock.^34,96^ This study utilizes the C_32_ homologue to avoid potential coelution issues.^97,98^ Using the interpretations in which 22S/(22S + 22R) ratios of 0.57–0.62 represent the equilibrium phase of the oil generation window,^34,63^ the samples analyzed, ranging from 0.59 to 0.64, are interpreted to have all been generated by thermally matured organic matter.

The moretanes/hopanes ratio is another metric of thermal maturity, typically measured using *m*/*z* 191 chromatograms, with C_29_ or C_30_ homologues.^34^ While C_30_ compounds are most commonly used, this ratio can also be determined using C_29_ compounds.^63^ Moretanes are less thermally stable than hopanes, and their relative abundance compared to that of hopanes decreases progressively with increasing thermal maturity. The moretane/hopane ratio decreases from approximately 0.8 in immature bitumens to less than 0.15 in mature source rocks and oils, reaching a minimum of 0.05.^63,66^ The moretane/hopane ratios of the samples analyzed ranged from 0.09 to 0.16 (Table 2), further supporting the thermal maturity of the organic matter that generated the samples analyzed.

The C_27_ 17α­(H)-trisnorhopane (Tm) is less stable than C_27_ 18α­(H)-trisnorhopane during catagenesis.^62^ As such, the Ts/(Ts + Tm) ratio, sometimes expressed as Ts/Tm, is widely used as a maturity proxy, though it is influenced by both source facies and thermal maturity.^34,99^ To gain a robust understanding of thermal maturity, the crossplot of the Ts/(Ts + Tm) ratio and C_29_ sterane 20S/(20S + 20R) ratio (Figure 12) was constructed, and it established that the samples were generated by source rock(s) composed of thermally mature organic matter.

**Figure 12:**
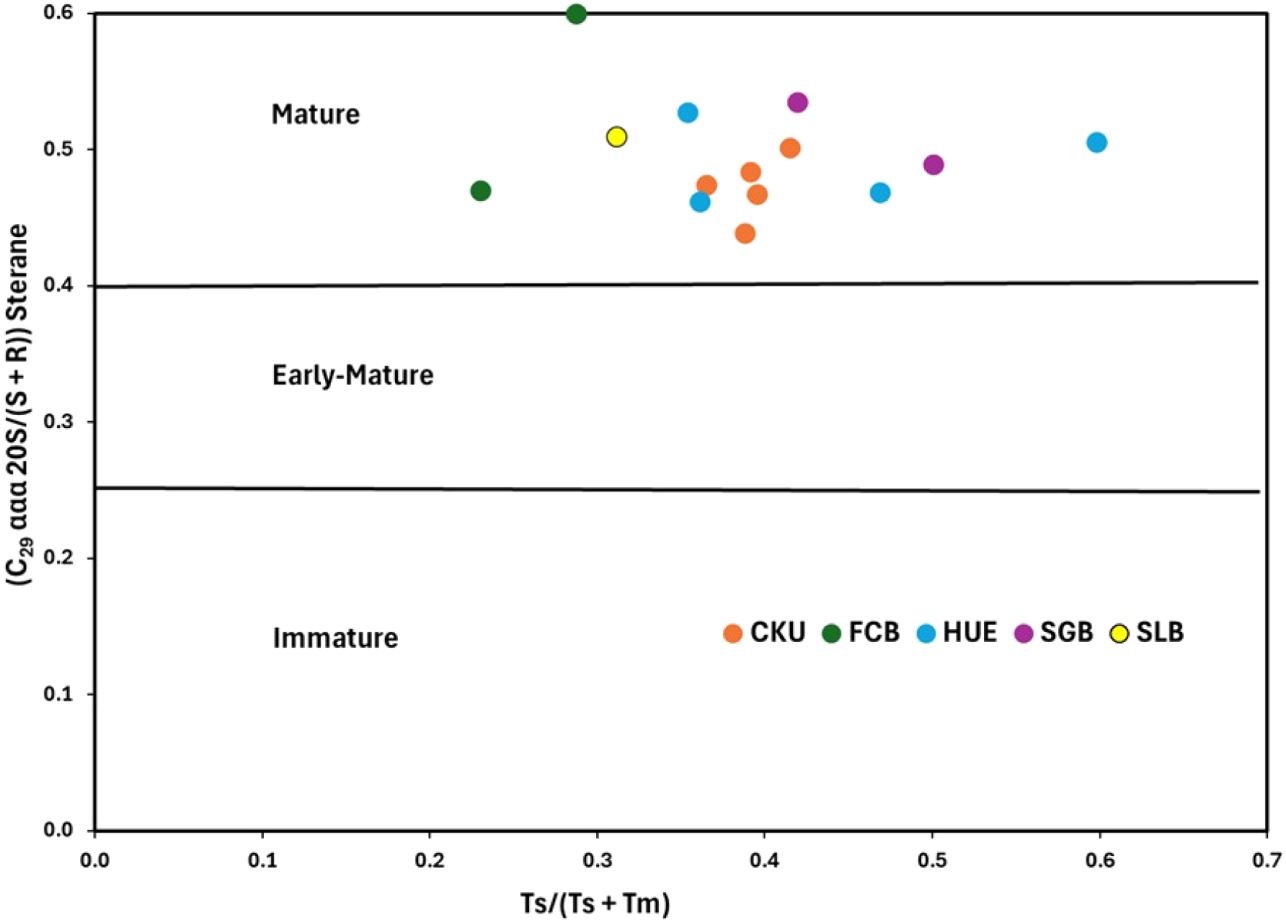
Crossplot of the C_29_ ααα sterane 20S/(20S + 20R) ratio and the Ts/(Ts + Tm) ratio, illustrating the maturity of the hydrocarbon samples.

## 6 Conclusions

This study provides a geochemical analysis and characterization of 18 hydrocarbon samples from five subbasins and six producing intervals across Kansas using GC and GC-MS techniques. The results of Pr/Ph ≥ 1, low NP/AK, CPI ≥ 1, cross-plot of DBT/PHEN ratio vs Pr/Ph, as well as C_29_/C_30_ hopane ratio <1 were interpreted to denote a shale lithology for the source rock that generated the hydrocarbons irrespective of their subbasin or producing intervals. Sterane distributions (C_27‑C29_), C_19_/C_23_ tricyclic terpane, and sterane/hopane suggest samples from the CKU, HUE, SLB, and SGB are predominantly marine-sourced organic matter, while FCB samples are terrigenous-sourced organic matter. The Pr/Ph ratios ranging from 0.98 to 2.25 and Pr/*n*-C_17_ vs Ph/*n*-C_18_ plot validate the inference of deposition in a marginal marine environment. The C_32_H/C_31_H hopane ratios (0.47 to 0.68) and the cross-plot of the Gam/(Gam + C_30_ Hopane) ratio vs Pr/Ph further support suboxic redox conditions with normal marine salinity conditions and no water column stratification. The thermal maturity of the source rock that generated the analyzed samples was interpreted to be within the oil generation window based on CPI and OEP values (∼1), C_32_ homohopane 22S/(22S + 22R) ratio ranging from 0.59 to 0.64, cross-plot of C_29_ ββ/(ββ + αα) vs C_29_ 20S/(20S + 20R) sterane, moretane/hopane ranging from 0.09 to 0.16, and plot of Ts/(Ts + Tm) ratio vs C_29_ sterane 20S/(20S + 20R) ratio.

Interpretation of the FCB subbasin is constrained by limited sample availability, as only two samples were analyzed. Although these samples consistently point to enhanced terrigenous organic matter input, the results should be considered preliminary, and extrapolation of the nature of the source rock for the FCB subbasin should be undertaken with caution until additional data become available.

On the basis of our findings, we recommend oil-source correlation analysis for future studies to definitively determine the source rocks that generated the hydrocarbons being produced in Kansas. The Chattanooga Shale found within Kansas and the Woodford Shale from the Anadarko Basin are recommended for future oil-source correlation studies.

This study advances our understanding of the Kansas petroleum system by emphasizing the geochemical signatures of its source rocks. Although the debate regarding the relative contributions of local vs external source rocks remains unresolved, the results prove that the hydrocarbons were generated by multiple source rocks. These findings provide a framework for future studies to consider both local and external sourcing as part of an integrated petroleum system model for Kansas.
